# Transforming tropical peatland governance to manage climate risks using the Three Horizons method

**DOI:** 10.1371/journal.pone.0324399

**Published:** 2025-11-20

**Authors:** Mark S. Reed, Dianna Kopansky, Sam Beechener, Alexa Green, Patrick Scheel, Ian Kendrick, Ifo Averti Suspense, Corneille Ewango, Euridice N. Honorio Coronado, Carlos Gabriel Hidalgo Pizango, Manuel Martin Brañas, Margarita del Aguila Villacorta, Haruni Krisnawati, Ioan Fazey, Susan Page, Lindsay C. Stringer, Carly Maynard, Johannes Kieft, Tom Curtis, Sian Allen, Montserrat Costa-Font, Rosie Everett, Emmanuel-Tsadok N. Mihaha

**Affiliations:** 1 Department of Rural Economy, Environment and Society, SRUC, Edinburgh, United Kingdom; 2 Global Peatlands Initiative, United Nations Environment Programme, United Nations Avenue, Gigiri Nairobi, Kenya; 3 University of Three Horizons, London, United Kingdom; 4 University of Marien N’Gouabi, Ave des Premiers Jeux Africains, Brazzaville, Republic of the Congo; 5 University of Kisangani, G57G+GGG, R408, Kisangani, Democratic Republic of the Congo; 6 Royal Botanic Gardens, Kew, Richmond, London; 7 Instituto de Investigaciones de la Amazonia Peruana, Iquitos, Peru; 8 Ministry of Forestry, Jakarta, Indonesia; 9 Department of Environment and Geography, University of York, York, United Kingdom; 10 School of Geography, Geology and the Environment, University of Leicester, Leicester, United Kingdom; 11 York Environmental Sustainability Institute (YESI), University of York, York, United Kingdom; 12 United Nations Environment Programme, Jakarta, Indonesia; 13 3Keel, Fenlock Court, Long Hanborough, United Kingdom; 14 Regional School of Water, University of Kinshasa (ERE), and Congo Basin Water Resources Research Centre (CRREBaC), Kinshasa XI, Mont Amba, Democratic Republic of Congo; National Research and Innovation Agency, INDONESIA

## Abstract

Tropical peatlands occupy at least 440,000 km^2^ and are estimated to store around 100 megatons of carbon, but are exposed to risks from both current and anticipated future changes in climate. Integrating knowledge from diverse sources using methods that can manage complexity is vital in order to identify transformational governance options for managing climate risks in these multifunctional social-ecological systems. This paper breaks new ground by applying the Three Horizons method to the governance of tropical peatlands. It evaluates the capacity for this method to generate transformative options that address conceptual and existential risks, as well as visible climate risks, whilst integrating research evidence with local knowledge. The paper focuses on countries that collectively represent the majority of global tropical peatland area and emissions, combining evidence from the literature with in-country expertise through Three Horizons workshops in Peru, Democratic Republic of Congo and Republic of Congo, and additional business-to-business engagement in Indonesia. The paper identifies a number of pathways that could transform the resilience of habitats and populations dependent on tropical peatlands, with community empowerment and payments for ecosystem services emerging as key themes across all four countries. Drawing on these findings, recommendations are made for managing climate risks through tropical peatland conservation, restoration and sustainable management. Application of the Three Horizons method demonstrates the critical role of integrating multiple knowledge sources to structure dialogues that can create credible and socially acceptable policy options for managing complex social-ecological systems.

## Introduction

Peatlands are the world’s largest terrestrial carbon store and important for the livelihoods of numerous rural and indigenous communities within or close to tropical peatlands [1]. Despite only covering 3–4% of the planet’s land area (ca. 500 million hectares), peatlands occur across all continents and are estimated to store up to a third of the world’s soil carbon (around 450,000–650,000 megatons); twice as much as the carbon stored in the world’s forest biomass [1]. However, degraded peatlands are a major source of greenhouse gases caused by human land use, deforestation, drainage and unsustainable use. Disturbed peatlands are both a cause of climate change, accounting for 4% of all anthropogenic emissions (excluding emissions from fire) and are affected by climate change, due to increased peat oxidation emissions associated with higher temperatures and more frequent and severe droughts [2–4]. There is an increased likelihood of severe peat fires on drained peatlands, with some estimates suggesting that when emissions from fire are included, peatland degradation may contribute as much as 10% of total global anthropogenic emissions [5].

Governance of tropical peatlands to manage climate risks is complicated by multiple factors including: limited awareness of peatland extent and condition, especially where they occur under forests; limited scientific knowledge of near- and longer-term climate risks and their impacts; limited awareness of appropriate approaches to climate change adaptation and mitigation; coordination and policy coherence issues across government departments and agencies (peatlands may be under the jurisdiction of forestry or agriculture depending on how they are used), semi-autonomous provinces and between countries; low visibility of peatlands in national policy making; resistance from groups with competing interests; and limited understanding of the social, economic and cultural impacts of decisions on local communities, corporations and others who depend upon peatlands [6]. Consequently, policy makers are making complex decisions with limited information about their likely environmental, social, economic and wider livelihood outcomes and environmental outcomes over both the short and long term.

Many ongoing efforts to tackle these governance challenges focus on finding ways to manage visible climate risks (e.g., technical challenges around restoration management, the creation of new livelihood options, or the management of financial risks in blended finance). Focus on visible risks may partly be due to the dominance of natural science assessments of climate risks in tropical peatlands, which has tended to overlook cross-sectoral social, economic and institutional challenges and opportunities, and the experiences and management of risk from the perspectives of the most affected populations [7,8]. This paper goes beyond the management of visible risks and investigates conceptual risks (e.g., the transformation of systems, structures, financial models and modes of governance) and existential risks (e.g., why the institutions that govern peatlands exist, and who should they serve) that typify the climate emergency [9].

New approaches, drawing on interdisciplinary and transdisciplinary methods including those from the social sciences, are needed to tackle the complexities arising from the visible, conceptual and existential risks arising from climate change in tropical peatlands. Inclusive and ethical engagement with local communities, corporations and the other human and more than human species that depend on peatlands, is essential to capture non-market values associated with ecosystem services, such as climate regulation and cultural identity, in decision-making processes [10,11]. Hence, it may be possible to identify approaches to managing conceptual and existential risks, as well as visible risks in ways that are adapted to the needs of the human populations and other species that depend upon peatlands [12–14].

Processes of transformational change are, however, dynamic and complex [15], typically taking place over extended time frames. They elicit multiple and likely contested perspectives around preferred outcomes and are unpredictable, leading to potentially unexpected, sometimes undesirable impacts [16]. To help scholars and practitioners navigate this complexity and identify transformation pathways, various futures methods have been proposed. For example, drawing on futures literature, the Futures Triangle [17–19] has a temporal emphasis to accommodate the so-called weight of the past, the push of the present and the pull of the future. With roots in the sociology of technology and evolutionary economics theory, socio-technical innovation (versions of this include Transitions Management and Strategic Niche Management) explores dynamic interactions between emerging niches, prevailing regimes and an overarching landscape to reflect transformative, non-linear social-technical change [20–24]. Meanwhile, social-ecological systems thinking (encompassing adaptive management and adaptive co-management) emphasises the evolving interdependencies between ecosystems and society across temporal and spatial scales, as well as social groups [25–27]. Despite calls to bridge these perspectives [28–30], the different theoretical bases for these approaches can make integration challenging in practice [31].

One approach that offers potential to combine the key strengths of each of these approaches is the Three Horizons method. In common with the Futures Triangle, Three Horizons visualises transformations as they unfold over time: the here and now, or business as usual (Horizon One); the emerging of the future (Horizon Two); and the turbulence and disruption associated with moving from the here and now (Horizon One) to the desired future (Horizon Three), which is embodied in Horizon Two [32]. Three Horizons thinking has been widely applied in a range of socio-economic and cultural contexts, to structure conversations across diverse perspectives (e.g., see [33] in southern Africa, [34] in the Canadian Arctic, and [35] in Northern Europe). In common with social-ecological systems approaches, Three Horizons provides a structured framework with the flexibility to accommodate diverse forms of knowledge, including that derived from social and natural science approaches, alongside local and other less formal forms of knowledge [36]. In common with socio-technical systems approaches, Three Horizons facilitates the identification and analysis of innovations in niches that could be mainstreamed in societal transformations that address visible, conceptual and existential risks in complex governance systems [32].

The overarching aim of this paper is to apply and evaluate the Three Horizons method to inform the transformation of tropical peatland governance. To the knowledge of the authors, it is the first time Three Horizons has been applied to peatland governance. The aim is achieved by:

- Assessing the potential for Three Horizons to generate transformative options that address conceptual and existential as well as visible climate risks, while integrating different kinds of knowledge; and
- Identifying novel governance options that could transform the resilience of habitats and populations depending on the peatlands of Peru, the Congo Basin and Indonesia; regions selected as focal countries for UNEP’s Global Peatlands Initiative that collectively represent the majority of tropical peatland area and emissions.

By examining these systemic climate risks, this paper attempts to move beyond risk management to identify innovations that could transform the governance of tropical peatlands for those (often vulnerable and marginalised) populations who are most exposed to these risks. The paper first provides background on the climate risks affecting tropical peatlands. It then combines top-down insights from an expert-driven global literature review with bottom-up insights from the comparative analysis of in-country surveys and workshops with people affected by climate change in peatlands. These insights are integrated using the Three Horizons method, an approach to dialogue that integrates evidence from the past with anticipatory and participatory forms of knowledge [32] to identify options that could transform peatland governance and reduce vulnerability to climate risks.

## Background: Tropical peatlands under threat

Tropical peatlands occupy at least 440,000 km^2^ and are estimated to store around 100,000 megatons of carbon [36,37]. Their greatest extent is in Southeast Asia, particularly on the coastal lowlands of western Indonesia, Malaysia and Brunei, in the Mekong delta, and on the island of New Guinea, both in Indonesia and Papua New Guinea, in the Congo basin of central Africa, and in Central and South America, with the most extensively documented systems found in the Amazon lowlands [36–38]. Holding some of the earth’s oldest peatland sites, (e.g., Borneo [39]) tropical peatlands also offer key insights into understanding of past human interactions as an archive of long term sociocultural and ecological heritage. Whilst an often forgotten tool in peatland conservation and engagement of communities [40,41], these archives can be the most informative for future management, protection and conservation [42–44].

Over millennia, these peatland ecosystems have functioned as net carbon sinks, having a cooling effect on the global climate. Since the 1980s, especially in Southeast Asia (and to an extent in Latin America), they have been subject to disturbance, drainage and land use change, in particular for industrial forestry and palm oil and smallholder farming. These systems now contribute significantly to anthropogenic greenhouse gas emissions [1]. Peatland disturbance has also led to an enhanced risk of peatland fire, a further source of greenhouse gas emissions, and has had major negative impacts on biodiversity, water supply and quality, human health and livelihoods [1,45]. Whilst tropical peatlands outside of Southeast Asia have been less impacted by anthropogenic disturbances, they are under increasing threat from a range of economic developments [46–48]. It has been estimated that conservation and restoration of tropical peatlands alone could reduce global greenhouse gas emissions by 800 Mt CO_2_e per year (close to 1.5% of annual global emissions). Conservation, sustainable management and restoration actions would also reduce exposure to subsidence leading to land loss, support biodiversity, improve water quality, reduce flood risk, reduce air pollution from peatland fires, protect important cultural heritage and maintain livelihoods [1,49]. As such, there is growing interest in the governance of tropical peatlands, to conserve and restore these systems to build resilience and manage climate risks [50] while maintaining the livelihoods of populations that depend on these ecosystems.

Tropical peatlands are exposed to risks from both current and anticipated future changes in climate [48,50,51]. Principal amongst these are the projected intensification of wet and dry seasons in Southeast Asia, in part driven by changes in the frequency, duration and intensity of droughts associated with ENSO (El Nino Southern Oscillation) and IOD (Indian Ocean Dipole) events [52,53]. Forecasts for the north-western Amazon basin of warmer temperatures and dry season droughts but increased precipitation and wet season flooding may protect these peatlands from climatic drying in the medium term [47]. In the Congo basin, models indicate a warming climate but no consensus on future precipitation levels [54,55]. Extended periods of reduced precipitation will negatively impact carbon sequestration and storage in intact tropical peatlands [56]. There are indications that recent ENSO-driven droughts in Southeast Asia are likely resulting in the loss of stored carbon from forested peatlands [57], whilst for the Congo peatlands, which are already at the lower end of the annual rainfall range for tropical peatlands, there is palaeo-ecological evidence that drier conditions can lead to an increase in peat decomposition rates and sizeable loss of carbon [58]. Increasing atmospheric CO_2_ concentrations represent a further potential risk to the stability of tropical peatlands. Whilst increasing levels of atmospheric CO_2_ may have a fertilization effect on peatland vegetation, with the potential for increases in forest productivity and rates of peatland carbon storage, changes in the seasonality of precipitation [59], in particular increased frequency and intensity of drought periods [60], could negate any such effects [61].

In peat landscapes that have been drained, the climate risks are further exacerbated by peat subsidence [62,63], an increasing likelihood of surface inundation during periods of high rainfall that will be further intensified by rising sea levels [64], and increased wildfire risks, creating irreversible impact to the health and livelihood assets of communities relying on these peat landscapes [65]. Risks are also exacerbated by an increase in anthropic ignition sources, which, combined with increased length and intensity of the dry season, increase the risk of fire, and can have impacts that extend beyond the peatland itself [45,66]. For communities living in or close to peatlands, and particularly for those dependent on the peatland ecosystem as a source of livelihood (through, e.g., fishing, farming, extraction of non-timber forest resources) these risks translate into challenges including potential declines in the populations of harvested wild species (e.g., fish, fruits, etc.) and potential loss of land due to subsidence and related productive assets; changing availability and management of water for smallholder farmers and plantation companies, with increasing risks of wet season flooding and dry season drought; and an increasing risk of fire, with a range of negative direct effects on livelihoods and health. Climate-related risks also exist for peatland restoration projects that aim to protect the remaining peat carbon store and restore natural vegetation cover whilst also ensuring livelihood security for local communities. These risks similarly extend to businesses dependent on the production of commodities from tropical peatlands, e.g., oil palm plantation companies.

## Methods

**Fig 1:**
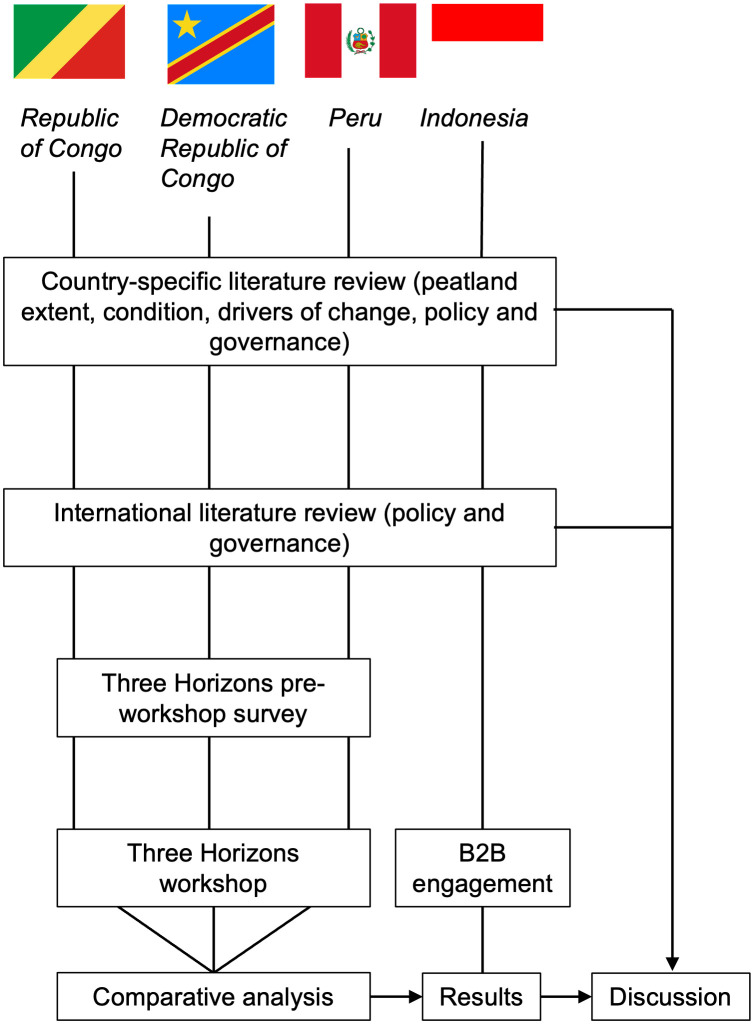
Research design, showing how literature review was integrated with empirical research in Peru, Democratic Republic of Congo and Republic of Congo, and insights from business-to-business (B2B) engagement in the Riau region of Indonesia.

An expert driven, narrative literature review of governance options in the context of tropical peatlands, was combined with in-country application of Three Horizons methods (see Three Horizons section below for details) in Peru, Democratic Republic of Congo and Republic of Congo, and business-to-business engagement in the Riau region of Indonesia (Fig 1). In Indonesia, it was not possible to apply the Three Horizons method directly because a research permit could not be secured within the timeframe of the project. Instead, we adapted our approach to elicit perspectives on future governance trajectories through structured dialogues with industry and policy actors. Indonesian engagement was designed to capture equivalent insights on near-, medium- and long-term governance challenges and opportunities, and findings were analysed within the same analytical framework used for the Three Horizons outputs, enabling integration with results from the other case studies.

Workshop teams in each country included literature review authors, enabling insights from the review to feed into the Three Horizon process alongside local knowledge from workshop participants. Country-specific review findings and international evidence from the review were used to interpret workshop findings (see discussion section), and generate conclusions of relevance to both focal countries and other comparable contexts. Bringing together top-down, expert knowledge with bottom-up, local expertise enabled a more comprehensive perspective on the challenges and opportunities for transformational change.

**Fig 2:**
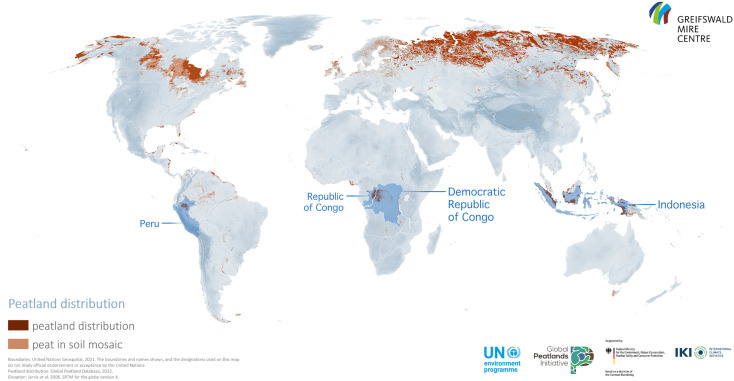
Distribution of global peatlands according to the Global Peatlands Assessment, showing the location of the study countries in blue (Source: Global Peatlands Assessment data retrieved from the Global Peatland Database compiled by the Greifswald Mire Centre. UNEP. Global Peatlands Assessment – The State of the World’s Peatlands: Evidence for Action Toward the Conservation, Restoration, and Sustainable Management of Peatlands. Main Report. Nairobi: United Nations Environment Programme; 2022. The map is based on data from the *Global Peatlands Assessment* and the *Global Peatland Database*, which is publicly accessible via the UNEP World Environment Situation Room: https://wesrmapportal.unep.org/portal/apps/experiencebuilder/experience/?id=33b49f8757c441faae975671c2425241).

The study focuses on peatlands in tropical regions, with particular emphasis on extensive peatlands recently described in the scientific literature in the Peruvian Amazon and Congo Basins [66–69]. Accordingly, the focus of the empirical research was the tropical peatlands of Peru, the Republic of Congo and the Democratic Republic of Congo (Fig 2). Reflections from business engagement around some of the innovations arising from this work in the Riau region of Indonesia were also integrated.

### Literature review

An expert-driven narrative literature review of governance options for peatland conservation, restoration and sustainable management in the context of climate change, was facilitated by UNEP’s Global Peatlands Initiative (GPI) as part of its Global Peatlands Assessment [1]. Authors were selected on the basis of their subject specialisms and to represent each of the main UN regions, whilst paying attention to gender balance and ensuring representation of early career researchers. Narrative reviews are expert-driven scholarly summaries that combine interpretation and critique [70,71]. Narrative reviews are better suited than systematic reviews for topics or questions where it is not possible to identify specific interventions or outcomes [71], as was the case for this work. Compared to systematic reviews, the expert-driven approach has the potential to miss important literature, but this risk was minimised by a rigorous and extensive review process as part of the production of the Global Peatlands Assessment. Key findings are summarised in Section 4 (for the full review, see [72]), and were used to supplement pre-workshop survey findings for discussion in workshops (described in the next section), in recognition that there may be relevant innovations in other countries internationally that could be discussed at the workshops, which may be relevant in each national context.

### Three horizons

**Fig 3:**
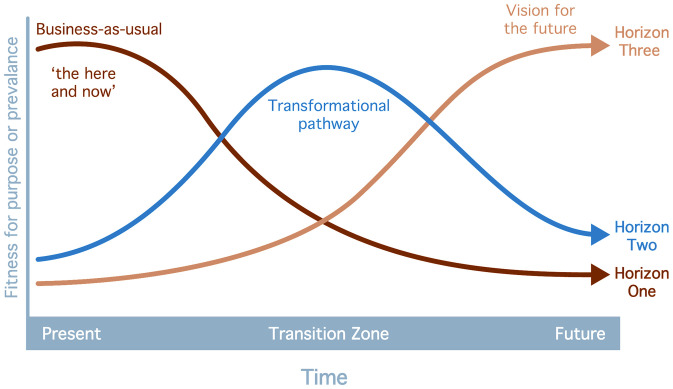
Visualisation of the Three Horizons framework (adapted from [32]).

In the Three Horizon workshops, actors from different disciplines, sectors and backgrounds were convened in dialogue, and combined evidence from the past and anticipatory forms of knowledge to map out how systemic pattern shifts could be facilitated [32]. The Three Horizons method structures dialogue about change across three temporal frames. Horizon 1 focuses on the present system, highlighting both its strengths and the problems that need to be addressed. Horizon 2 captures transitional activities, including emerging innovations and contested practices that signal movement away from the current system. Horizon 3 represents longer-term aspirations for fundamentally different ways of working (Fig 3). Facilitated workshops used these three horizons as prompts to enable participants to articulate immediate challenges, identify near-term options, and consider transformative pathways over the longer term.

In close consultation with in-country collaborators, initial discussions sought to identify a shortlist of 15–20 relevant parties (in each country) with a view to recruiting 6–8 participants from each country to the project. To ensure participants were as representative and diverse as possible, they were selected on the basis of: i) relevance to the goals of the project (based on their level of interest, degree of influence and impact on them of project outcomes; [73]); ii) sectoral interests (public or private sector, academia, NGO, community); iii) balance of interests between environment, economic and social priorities; and iv) organisational level (individual, group or organisations). Prior, informed, written consent was obtained from all survey respondents and workshop participants. Guided by local partners, this was based on the principles that:

- the purpose of the research was clearly understood;
- participation was voluntary with a right to withdraw, without explanation, at any stage;
- findings would inform reports to the funders and for wider publication;
- any quotes used for illustration would not be attributed to individuals;
- the anonymity of participants would be respected; and
- workshop discussions would be recorded for analysis.

Those participating from DRC and Republic of Congo were formally educated stakeholders representing the interests of the organisations they worked for (not members of local communities), and where local communities were engaged in online workshops in Peru, this was managed by local co-authors from IIAP, who maintain long-standing working relationships with these communities.

A letter of introduction and introductory video setting out the background to the project and anticipated terms of engagement was agreed with in-country collaborators and translated from English into Spanish and French. An initial survey was sent with the letter of introduction and video on 22^nd^ November 2021, to identify challenges and opportunities for tropical peatlands in their region and more widely (the survey closed on 21^st^ January 2022). Prior permission for the survey was granted by SRUC’s Social Science Ethics Committee (ref. 21–42556052). The survey consisted of open questions structured around the here and now (Horizon 1), the desired future (Horizon 3) and potential for change (Horizon 2):

- Horizon 1
  - What shows peatlands are under strain?
  - What suggests tensions are increasing for peatlands, related to consequences for planetary health?
  - What current aspects can/must be maintained or amplified for future peatland conservation and management?
- Horizon 3
  - In 2031, what would indicate that peatlands are fulfilling the needs of society?
  - In 2031, what would indicate that peatlands are well aligned with the current and emergent needs for planetary health?
- Horizon 2
  - What aspects of the envisioned peatlands future (H3) are already present?
  - What existing initiatives could enable the transition to Horizon 3?
  - What initiatives are needed to enable the transition to Horizon 3?

**Fig 4:**
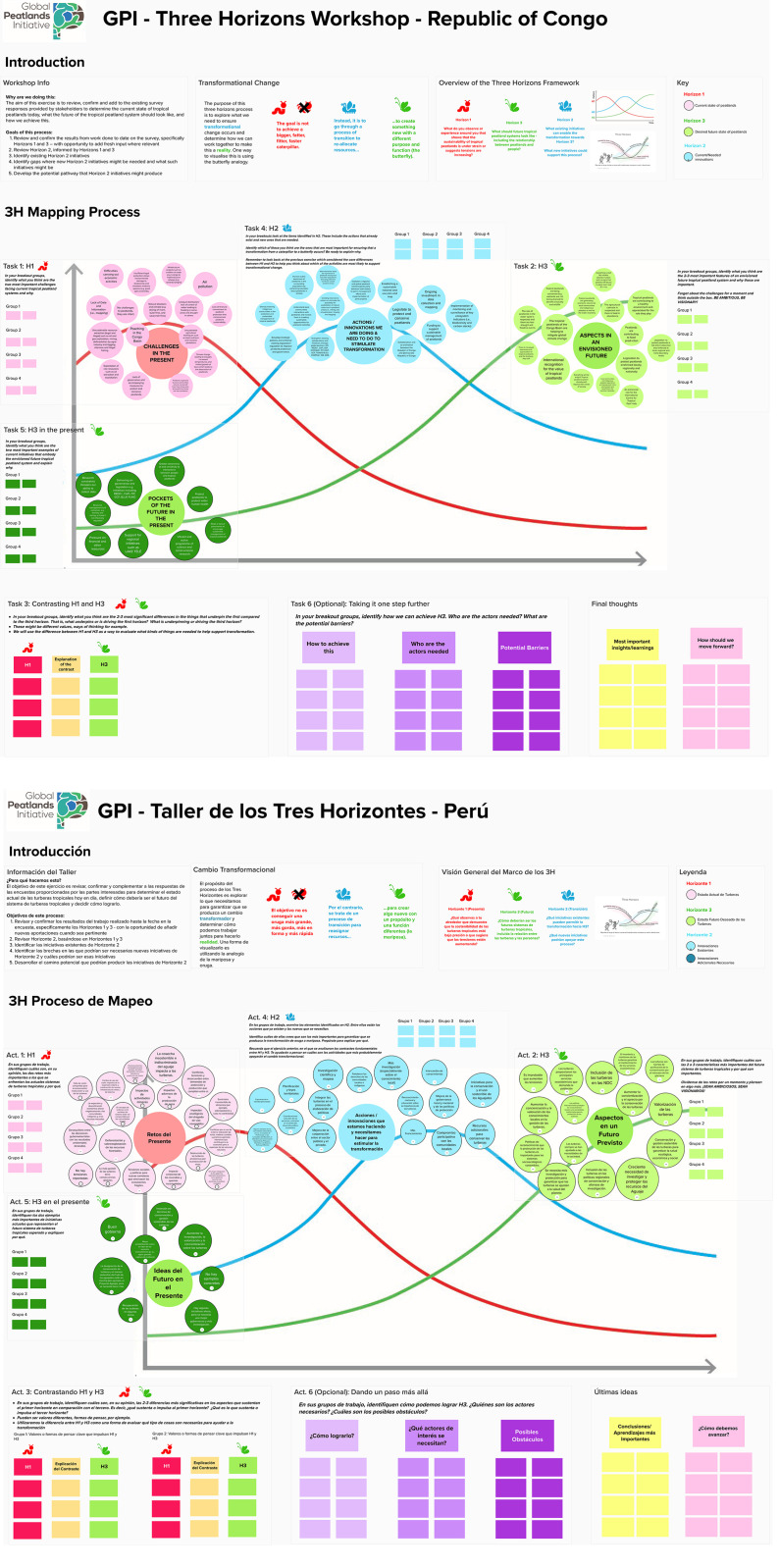
Continued.

A total of 10, 16 and 11 completed responses were received in Peru, Republic of Congo and Democratic Republic of Congo respectively, and translated into English, with translations sense-checked with in-country partners to ensure accuracy. Qualitative analysis was used to identify themes from the open-ended question data, assigned to horizons based on the section of the survey answers appeared in, as these were organised by horizon. These were visualised along each of the three horizons, as illustrated in Fig 4 (from the Republic of Congo workshop), for use in workshops, integrating insights from the literature review where relevant.

Participants were then invited to discuss content and placement of material in workshops. Social science ethical approval was granted by SRUC’s Social Science Ethics Committee for the online workshop in Peru (ref. 62–48327985) and verbal, informed consent was obtained from participants at the workshop. SRUC’s Social Science Ethics Committee also gave prior approval to the in-person workshops in Democratic Republic of Congo and Republic of Congo (ref. 62–48327985) and written, informed consent was obtained from participants in these workshops. Two half-day virtual workshops in Spanish were held in Peru on 27 and 28 June 2022 with 12 participants, facilitated from the UK with English translation. One full day, in-person workshop was held on 28 July 2022 in Brazzaville, Republic of Congo, with 17 participants, facilitated locally in French with live English translation via zoom to the UK. One full day, in-person workshop was held on 23 March 2023 in Kinshasa, Democratic Republic of Congo, with 18 participants, facilitated locally in French without either English translation or zoom link to the UK. All notes from workshops were translated into English for analysis, and the translation and analysis were subsequently discussed/validated/sense-checked with in-country collaborators.

Workshops followed the five steps identified by [32] to put Three Horizons thinking into practice. Discussions began with the present situation in Horizon 1, envisioning an idealised future in Horizon 3 before back-casting to the transitional Horizon 2. This is similar to Theory of Change methods, in which back-casting is the principal method through which intermediate actions are identified to move from the present to the future [74]. As such, Step 1 began by clarifying concerns in the here and now, for example tensions and mismatches between current practice and the wider, evolving context which were identified in the interviews. Step 2 shifted the focus to the future, by refining the aspirations for change. Step 3 acknowledged the oft-blurred boundaries between the present and the future and identified so-called ‘pockets of the future’ already present at the “margins of the mainstream” [32, page 46]. Step 4 addressed the pathway to change, prompting consideration of all that will be involved in realising the transition from the here and now to a desired future. Step 5 captured those features of the present that may usefully be sustained to support the process of change. No further formal analysis was conducted on workshop materials, which were translated to English before being summarised under each Horizon for the results section which follows.

Although it was not possible to get a research permit to extend the Three Horizons research to Indonesia, business-to-business engagement was facilitated via project partner 3Keel with a range of Indonesian businesses affected by peatland subsidence due to drainage [75,76], to explore the feasibility of ideas emerging from research in other countries around landscape-scale ecosystem markets. Activity focussed on the Riau region, which due to an increasing demand for peatland commodities in recent years, and the associated commercial interests, has faced significant issues with land subsidence, as peatlands have been drained for timber production and plantations producing palm oil and rubber. Subsidence driven flood risk is likely to be exacerbated by climate change, which is responsible for both rising sea levels and increasing intensity of rainfall. The region is dominated by a handful of large international commodity traders who source from a combination of industrial plantations and small-scale smallholder farmers. 3Keel conducted interviews with four companies operating within palm oil and pulp, paper and packaging supply chains and purchasing from both large and small scale production units, as well as an industry initiative supporting landscape projects within Indonesia. Interviews focussed on four key questions:

1. What is the level of knowledge of risk from peatland degradation in Indonesia and Riau, Sumatra within companies operating in the region?
2. What actions are being taken to address this risk?
3. Have any actions been particularly effective to date? If not, what have been the barriers to effective action?
4. Is there a desire for an intervention that combines different sectors and industry actors to address risks from peatland degradation?

## Results

### Review of policy options

A narrative review of existing policies identified a range of policy and governance instruments, and broader enabling conditions, that could halt further degradation of tropical peatlands, and facilitate their conservation, restoration and sustainable management. Table 1 summarises the regulatory, financial and market, and co-management policy and governance mechanisms identified in the review. A growing number of these are now being implemented by governments around the world, with evidence of new policies and strategies to protect, restore and sustainably manage peatlands in at least 23 countries, which together are responsible for over half of global peatland emissions [6]. A full review of the global policy and governance options made by [72] are summarised in [77].

**Table 1:** Policy and governance options for conserving, restoring and sustainably managing peatlands identified from the literature by [72].

Policy or governance option	Summary
Regulatory controls	
Protected areas	Peatland ecosystems across the globe remain largely unprotected from infrastructure development, mining, oil extraction, and conversion to agricultural use and therefore are highly vulnerable to land use change. Where protected peatlands are intact and subject to minimal disturbance, these areas tend to be small remnant patches in an otherwise disturbed and managed landscape. Where more extensive areas of high-integrity peatlands are protected, their extent is typically limited in comparison to the extent of surrounding unprotected areas, and there are rarely sufficient resources for effective conservation. Where sites have been impacted by drainage, logging, fire, invasive species or other forms of exploitation or disturbance, they may need active interventions to conserve or restore their key features and functions, and this can be resource intensive. In addition to protected areas designated by governments such as national parks, nature reserves and wildlife sanctuaries, etc., peatlands are also conserved within the lands and territories of Indigenous Peoples and Local Communities (IPLCs). When overlaps with other protected areas are excluded, IPLC lands in good ecological condition cover 17.5% of the world’s terrestrial surface.
Buffer and managed utilisation zones	Peatlands outside of formally designated protected areas may be afforded some level of statutory protection if they form part of planning zones, as outlined in Indonesian Government Regulation 57/2016, amending regulation 71/2014 concerning protection and management of peat ecosystems, which includes buffer and utilisation zones permitting a wider range of activities. These zones are increasingly common around protected areas, providing access for certain uses to local communities, provided that strict sustainable management requirements are followed. In many peatlands, this includes wetland buffer zones to filter out nutrients from surrounding agricultural land. For example, the EU Water Framework Directive (2000) highlights the importance of peatlands as ‘buffer habitats’ for water purification, which have to be taken into account in River Basin Management Plans.
Regulation to restrict or ban extraction or other damaging activities	Regardless of their statutory protected status, states may decide to impose moratoria, regulations and controls. Licensing may be aimed at preventing or eliminating damage to the site’s intrinsic values, or to limiting fire risk, carbon loss or off-site pollution (e.g., of waterways) arising from some form of peatland land use (e.g., peat cutting, drainage, tree planting, fire use, etc.). For example, in 2011, the government of Indonesia suspended all new concessions for conversion of peatland and primary forest areas to other uses (namely, oil palm, pulpwood and logging concessions) in line with the country’s commitment to reduce greenhouse gas emissions and adapt to climate change. The moratorium was implemented in 2011 on a temporary, two-year basis. It was subsequently renewed on several occasions and then made permanent in 2018. The effectiveness of the moratorium and other regulations aimed at preventing peatland degradation has been the subject of some debate. Several studies have highlighted limited effectiveness in reducing forest loss, drainage and forest fires, while others have concluded that the moratorium led to a reduced rate of peatland conversion.
Financial and market instruments	
Public funding for restoration, conservation and sustainable management	To protect intact peatlands, restore damaged peatlands and pay for sustainable practices that deliver goods, public funding may be offered to peatland owners and managers. However, few governments have sufficient funding to meet the scale of conservation and restoration needed, even when conservation designations are in place. Conservation and restoration of tropical peatlands alone could cost $40 billion US Dollars but could reduce global greenhouse gas emissions by 800 Mt CO_2_e per year (close to 1.5% of annual global emissions), and could generate other valuable benefits and savings. For example, it has been estimated that peatland restoration in Scotland could deliver a saving of £191 million British pounds per year whilst providing a range of co-benefits such as the provision of drinking water. In recognition of the many valuable public benefits provided by peatlands, a growing number of countries are introducing peatland policies and strategies, many of which include public funding for peatland conservation, restoration and sustainable management [6].
Removing policies that lead to perverse outcomes for peatlands	Subsidies may exist for “land improvement” that promote the drainage of peatlands in an attempt to improve the productivity of peat soils, often with the addition of soil amendments, typically leading to significant emissions via oxidation of drained peats and erosion by wind and water after peat soils are brought into cultivation. Alternatively, a lack of clear policy definitions for peatlands may lead to policies that promote inappropriate management of peatlands. For example, in Chile, peat and its overlying vegetation were considered as separate products for which exploitation was regulated by different government entities. This led to a contradiction between the Mining Code, operated by the Ministry of Mining, which prevented the exploitation of peat as a fossil resource, and Ministry of Agriculture regulations, which managed *Sphagnum magellanicum* as a non-wood forest product that can be exploited and commercialized under regulation. As a consequence, to protect peatlands in Chile it was first necessary to legally define the peatland as an ecosystem where vegetation and peat are inherently associated.
Subsidising paludiculture	Subsidising paludiculture (the cultivation of crops on wet or rewetted peatland), for peatlands that are already in use for agriculture, has the potential to make a significant impact on the achievement of climate and biodiversity goals while providing income for farmers who have previously practised drainage-based agriculture through the production of high-quality fibres and biomass for a growing bio-economy. Subsidies are necessary due to the opportunity costs of switching from higher value arable or horticultural crops. In the EU Common Agricultural Policy, paludiculture was largely excluded from receiving payments for development, maintenance or expansion of agricultural activity. This contradiction has been resolved in the next funding period (2023–2027), where paludiculture will qualify for funding, including specific conditions to preserve peatlands. Germany recently started four large scale paludiculture projects with a duration of 10 years and with total funding of €48 million Euros to showcase and develop different paludiculture practices.
Taxation	Taxes may be introduced to reduce pressures on peatlands and to generate revenue, for example there are proposals for a “carbon emissions land tax” in Scotland, based on the polluter pays principle, taxing landowners that leave peatlands in a degraded condition, as an incentive to accept public subsidies or work with carbon markets to put land under restoration management [79]. Taxes could be introduced in order to further reduce the likelihood of damage and reinvest tax revenues into restoration and community initiatives.
Carbon and other ecosystem markets	Markets for carbon, biodiversity, water quality, flood risk and a range of other ecosystem services are proliferating rapidly around the world, including international compliance markets (Article 6, Paris Agreement), international voluntary markets (e.g., Gold Standard, Verra) and national/sub-national compliance (e.g., cap and trade or emissions trading schemes in California and the EU) and voluntary markets (e.g., Germany’s MoorFutures, the UK’s Peatland Code and max.moor in Switzerland). Where international voluntary markets are not financially viable, of sufficient integrity or sufficiently adapted to national contexts, it is possible to create new domestic markets to channel private investment into peatland restoration and management.
Blending public funding with private finance	Ecosystem market finance may be integrated with public funding to de-risk and leverage additional private investment, increasing the overall level of funding available for peatland conservation, restoration and sustainable management. Options include: i) full public-private co-procurement of public goods, in which public and private finance are integrated into a single fund at a landscape scale designed to deliver multiple outcomes; ii) co-ordinated public-private funding of public goods, delineated in space or time, enabling the market to pay for as much as possible, while public payments focus on market failures and those who are not prepared to accept private finance; and iii) Business as usual, whereby private funding pays for services that are not already being procured by public funding, with limited coordination (this is the scenario in most countries).
Co-management approaches	
Building capacity for co-management approaches to peatland management	The complexity of peatland social-ecological systems requires multi-level governance that empowers multiple groups (including those who are currently disempowered and marginalised), with different knowledges and values, to engage at a range of spatial and governance scales. Participatory and co-management approaches have been developed to support the coordination of these sorts of governance processes, allowing for the incorporation of diverse values in decision-making processes. This is particularly important when trying to understand and negotiate potential conflicts that may arise between rights-holders and other parties as a result of management interventions, like restoration. Coordination can be enhanced via “boundary organisations” that help communicating, translating, and mediating between different knowledge systems.
Behaviour change initiatives	Behaviour change initiatives may be able to further support changes in peatland use and management. Human behaviour follows predictable patterns and theories of behaviour change, such as the Theory of Planned Behaviour and social practice theory, which can be used to understand and influence environmental decisions. Such approaches need to be sensitive to social, cultural, political, and historical contexts, for example there is evidence that religious leaders have helped trigger peatland restoration in Indonesia.
Engaging diverse worldviews, values and people	It is important to recognise and address power asymmetries and diverse values in decision-making processes to achieve more equitable and sustainable policy outcomes. This needs to consider factors like gender and age, which impact resource distribution, engagement levels, and leadership opportunities. Collaboration among conservationists, industrialists, policymakers, and local/Indigenous communities is crucial but can lead to mismatches in perspectives unless all parties are involved from the beginning. In particular, it is important to encourage and acknowledge the contributions of women and other marginalised groups in peatland decision-making processes, addressing their unique perspectives, challenges, and barriers.
Recognising, valuing and integrating diverse knowledge systems	Community knowledge systems, perspectives, priorities, and values need to be integrated in peatland conservation and management. Local ecological knowledge includes observational knowledge, practical experience, and the beliefs of individuals. Integrating different forms of knowledge, including Indigenous and traditional ecological knowledge, can provide a more comprehensive understanding of long-term environmental changes. Various frameworks and research approaches, such as mixed methods and interdisciplinary approaches, storytelling, arts-based methods, and critical ethnography, can facilitate the inclusion of different ways of knowing, challenge biases and assumptions around evidence and truth, and facilitate unlearning and the reimagining of how we produce and value knowledge.

The review process and broader global priorities [77] highlighted that whilst obvious, priority should be given to protecting peatlands from being converted, drained and/or modified [78]. For tropical peatlands, activities that continue to drain and degrade peatlands need to be phased out. Damaged peatlands need to be rewetted and restored to conserve carbon stores and enable the recovery of ecosystem services. Subsidies that incentivise practices that degrade or convert peatlands to other land uses (e.g., certain agricultural activities, forestry and mining) need to be identified and removed or changed, where possible redirecting savings to pay for and incentivise restoration of degraded sites.

Where relevant, evidence from the narrative review was integrated with pre-workshop survey findings and discussed by participants during in-country workshops. The international policy and governance options described in this section, usefully frame the insights from national workshops, which are presented in the next section. Both national and international insights are then discussed in the subsequent section, considering how recommendations may play out across different time and spatial scales, as well as across different social groups and cultures.

### Empirical findings

Findings in this section are sourced from the in-country workshops and presented in the order in which the Horizons were discussed (1-3-2). The language is either directly quoted or paraphrased from the discussions (direct extracts from workshops are represented in quotation marks). Table 2 summarises the key findings from each workshop.

**Table 2:** Summary of findings from the three workshops.

	*Challenges of the present*	*Vision for the future*
*Republic of Congo*	Lack of legislation and governance. Lack of financial resources to conserve peatlands. Unsustainable anthropogenic activities.	The tropical peatlands of the Congo Basin are helping to mitigate global climate change. Legislation to protect peatlands is not only in place but also enforced at local, regional and trans-boundary levels. There is now mapping, awareness raising, data collection and monitoring. An integrated approach is being implemented that brings together peatland conservation, governance, management, funding and legislation. There is a role for the International Tropical Peatlands Centre as they play an increasingly important role in funding peatland management.
*Democratic Republic of Congo*	Lack of governance and legislation. Unsustainable use of resources in some peatlands. Scientific evidence to guide decision making, and cooperation for transboundary management of the shared ecosystem between Republic of Congo and the Democratic Republic of the Congo.	The government works closely with local communities to implement a national strategic plan for peatlands. Knowledge exchange enhances local communities’ understanding of the global value of peatlands and informs the scientific community of sustainable peatland practices and cultural value. The Congo Basin becomes a hub for peatland ecosystem biodiversity research and enhances eco-tourism opportunities.
*Peru*	Lack of legislation and governance. Unsustainable use of resources. Commercial agriculture. New infrastructure projects.	Peatlands in all their forms are embedded in the global consciousness and valued for their local and international ecosystem services. Practice of good governance to protect peatland resources in the Amazonian and Andean regions through legal measures. More knowledge about peatlands is shared locally and nationally which increases its value. Local communities are economically and financially supported to ensure sustainable management.

#### Republic of Congo.

***Horizon 1 Findings:*** Horizon 1 describes the current situation in the Republic of Congo (RoC) peatlands. The main issues affecting peatlands in the RoC were related to governance and legislation, lack of resources to conserve peatlands, and unsustainable anthropogenic activities (Table 2). Although there were laws pertaining to environmental protection and protected areas, there were no specific legal protections for wetlands or peatlands in RoC beyond Ramsar designations, no agreed national definition of peatlands, and no other relevant legislation (e.g., conservation law) that could be used to protect important peatlands. Peatlands were managed under different laws, e.g., laws for the protection of forests or indigenous communities. For example, under the 2008 Law for Species there are provisions for relevant parties to have access and harvesting rights and there is a separate framework for forests. There may also be multiple overlapping protected areas, where a designated protected area (e.g., a reserve or a park) may also contain wetlands protected under the Ramsar Convention, e.g., Lac Tele.

Participants highlighted that it is difficult to protect and conserve peatlands without sufficient accompanying measures such as data showing the location and condition of peatland areas. There was also a need for specialised public and private institutions and financial resources dedicated to peatland conservation. Unsustainable anthropogenic activities in and around peatland areas were a threat in some locations, including slash and burn agricultural techniques, industrial plantations, timber extraction and illegal fishing.

Peatlands in the RoC were also affected by climate change, causing some peatlands to dry more and others to flood more, while increased temperatures, which combined with deforestation, were leading to drying out and oxidation, or erosion of peatlands. Oil and gas exploration and extraction were additional potential issues affecting peatlands although there was some debate as to how affected peatlands would be by these activities. Illegal activities like poaching were a threat to the ecology of RoC peatlands, but these ecosystems also provided opportunities for a range of legitimate and sustainable economic uses, which are considered next.

***Horizon 3 Findings:*** Horizon 3 represents the vision for the future of RoC peatlands, as articulated by workshop participants. The consensus was that the future for the RoC peatlands was positive. In their vision for the future of RoC peatlands, workshop participants envisioned:

- Peatlands are better understood and are “helping to balance the global climate”.
- Diverse groups of relevant parties are coming together with a shared interest in their protection.
- There is a commitment to collecting information, in all its forms, that reflects the full diversity of these complex ecosystems and the changes they are undergoing.
- By bringing together scientific data and local knowledge, “each informs the other to give the best possible all-round perspective”. Around the world, peatlands, including the tropical peatlands of the RoC, are helping to mitigate climate change. Their role is appreciated by the international community.
- Attributing an economic value to this function helps to protect them against unsustainable development and acts as a counter-balance to the ongoing pressure for resource extraction.
- When it comes to peatlands, there are many interested parties. For some, the peatlands are their home; for others, they are their place of work or a potential source of revenue. A joined-up approach to decision-making sees the participation of all, including: civil society such as local NGOs specialising in the preservation of peatlands, as well as international bodies such as the ITPC; representatives of the relevant public administrations, including the Ministry of the Environment, Forest Economy, Land Management, and others; actors with a role in law enforcement such as the police, water and forestry agents.
- There is close co-operation with the neighbouring Democratic Republic of Congo. But, perhaps most crucially, relevant parties are embedded at the heart of the decision-making process to ensure their voice is heard.
- To support the above, governance systems enable a diverse range of relevant voices to be heard. Enabling legislation is in place, and enforced, to ensure the peatlands of the RoC are sustained. They, in turn, are sustaining populations as well as contributing to wider planetary health.

***Horizon 2 Findings:*** Horizon 2 describes the transformational pathway through which RoC peatlands move from their current status (Horizon 1) to their envisioned future (Horizon 3). Workshop participants identified a number of initiatives underway, helping support a transition to their desired future:

- Currently there is ongoing investment in data collection and mapping by academia.
- There is also funding to support sustainable management of peatlands including a project on forest and wildlife management in peatland areas.
- Awareness of the importance and threats to peatlands is being raised through national media and online videos.
- There is a strong collaboration and coordination between the RoC and DRC which can be enhanced by establishing a regional and global peatland network.

To stimulate transformation however, participants identified several actions that need to be taken:

- Efforts to protect peatland ecosystems could benefit from enhanced technical knowledge through national land use plans and maps, implementation of monitoring and surveillance of key ecosystem indicators, and increasing funding for all levels of research.
- It may also be necessary to strengthen existing collaborations and initiatives amongst national and global partners, and relevant parties must be involved in the protection and sustainable management of peatlands.

#### Democratic Republic of Congo.

***Horizon 1 Findings:*** Horizon 1 describes the current situation in Democratic Republic of Congo (DRC) peatlands. All land and nature (including flora and fauna) are legally owned by the government in DRC. However, certain groups have the right to exploit the land and many communities are still in the process of claiming their land back from colonial times. The government has some control over these lands, particularly for the development of national infrastructure. Now that the government sees the wider global economic value of tropical peatlands, tensions are rising regarding who has ownership and rights to the land, and for what purposes.

One of the main challenges facing peatlands in DRC is the impact of extractive industries such as artisanal logging and oil and gas exploration. Oil and gas exploration is a particular challenge for the protection of peatlands because the government sees this industry as a significant opportunity for economic development in the DRC particularly with regard to the Nationally Determined Contributions (NDC) and the achievement of the SDGs. Indeed, as a party to the UNFCCC, the DRC is committed to limiting the global temperature increase to 1.5°C, with a 21% greenhouse gas emission reduction target by 2021. However, the implementation of these international commitments will require significant financial resources, estimated at US$48.68 billion for the period 2021–2030 in the NDC. The DRC is also working to update its National Biodiversity Strategies for Sustainable Management (NBSAPs) in line with the new Kunming-Montreal global biodiversity framework, which replaces the Convention on Biological Diversity.

***Horizon 3 Findings:*** Horizon 3 represents the vision for the future for DRC peatlands, as articulated by workshop participants. In this envisioned future:

- The DRC government works closely with the communities that live in and manage the peatlands. Through the implementation of a national strategic plan, the value of tropical peatlands is formalised at both the local and national level, giving communities a platform and ability to define their interests to ensure the protection of their rights. The government respects communities’ right to the land and takes their lead on how peatlands should be managed. The government continues to subsidise the communities to help them to manage the land properly.
- Local communities continue to value peatlands for their immediate benefits but also understand the global implications of peatland management and particularly how they affect global carbon cycles and how they might benefit directly themselves from carbon markets.
- Local communities’ knowledge of climate change and carbon markets and the wider implications of sustainable peatland management is enhanced through knowledge exchange opportunities. As the carbon market expands, this is particularly important as local communities “will begin to see their international significance and power”. Through this enhanced understanding, local communities are better informed to make decisions about land use practices which benefit themselves and the global community.
- The Congo Basin will also become a hub for research on peatland biodiversity. In addition to remote sensing and mapping, there is enhanced research on the flora and fauna of these tropical peatland ecosystems. Researchers from local universities are given more financial support to conduct this research which also leads to enhanced expertise in this field. Socio-cultural practices and the ecosystem services provided by peatlands are further understood and appreciated, “and can be factored into other natural capital markets”. This can also eventually bring in eco-tourism opportunities “for bird watchers or other nature enthusiasts”.

***Horizon 2 Findings:*** Horizon 2 describes the transformational pathway through which DRC peatlands move from their current status (Horizon 1) to their envisioned future (Horizon 3). Participants suggested that:

- The government should first implement a land use management plan specifically covering protection for peatlands, before considering any further oil exploration in the Congo Basin. The role of local communities in peatland management was also discussed. Workshop participants called for a land use plan for peatlands which outlines a key role for local populations to play in continuing to manage these peatlands because they have been doing so successfully for many years.
- Participants called for land reform, granting tenure rights to local communities as part of a national strategic plan for peatland management similar to existing community forest law. It was argued that this was particularly important in light of carbon markets expanding into DRC, in particular REDD+ initiatives which have been proposed to protect peatlands from deforestation, to avoid what was described as the potential for a “wild west scenario”. In this context, the cultivation of malanga (*Xanthosoma sagittifolium*) has been promoted to support local communities living in peatland resulting from the permanent flooding of areas in the Cuvette centrale of the DRC, such as Bomongo, Makanza, Lukolela, Bikoro, Kungu, Kiri, Oshwe, Kutu, etc.
- Any national strategic plan must be developed from the bottom up, involving local communities, including forested peatlands community leaders, as well as relevant national and international organizations. In the Congolese context, peatland management is emerging as a new area of natural resource governance, leading to crucial decisions on its inclusion in international REDD+ programs or the creation of similar national programs. Since 2012, the DRC has been implementing a national REDD+ strategy that aims to reduce greenhouse gas emissions by conserving forests and increasing forest carbon stocks. As carbon reservoirs, Congolese tropical forested peatlands play a key role in this strategy, storing large amounts of organic carbon over long periods of time. Their conservation thus contributes to reducing CO2 emissions and combating climate change, while preserving their capacity to regulate the global climate in the long term. The inclusion of forested peatlands in the REDD+ strategy strengthens the DRC’s efforts to mitigate climate change and protect the biodiversity associated with these ecosystems.
- It was argued that carbon markets could become an increasingly important opportunity to fund sustainable management practices, leading to the long-term protection of peatlands. One issue was that local communities did not have “large enough swathes of land to count for the carbon market trading scheme”. Participants suggested a campaign “to connect the communities’ lands under one forest reserve to ensure its protection against extractive anthropogenic activities”.
- Although there is limited farming in the peatlands of DRC, unsustainable forestry practices remain an issue. Participants therefore called on the international community to help fund alternative economic opportunities, perhaps through the carbon market, to reduce reliance on extractive industries and provide local communities with compensation for the sustainable management of peatlands.
- An information campaign using simple terms to explain to local communities the global implications of their practices on climate change and other ecosystem services would be beneficial.
- Participants emphasized the urgent need for a better scientific understanding of peatlands in DRC. Despite the many ecosystem services they provide to humanity, in-depth knowledge of their current functioning and future role in the context of climate change is still limited. To fill this scientific gap, there were calls for projects to foster interdisciplinary research collaborations. These initiatives would bring together researchers from around the world and from the DRC, with the aim of stimulating scientific investment in this under-explored region and developing local human and technical capacity. In-depth studies on biodiversity and the quantification of ecosystem services in the peatlands of the Congo Basin were therefore deemed essential. Given the value of these ecosystem services, it was hoped that this would translate into increased financial support for local universities and students who wished to devote themselves to the study of peatlands.
- In response to the Kunming-Montreal Global Biodiversity Framework (30x30 target), DRC plans to expand the area dedicated to conservation by strengthening the Other Effective Conservation Area. Discussions highlighted the possibility of designating certain peatlands as protected areas, which would play a key role in preserving these important ecosystems for future generations. As unique and valuable ecosystems, peatlands are home to remarkable biodiversity and play a key role in climate regulation, justifying special protection from unsustainable human activities. However, the establishment of these protected areas requires collaboration between stakeholders, including local communities, provincial governments, central administrations at the national level, the private sector, civil society and international organisations, and must be accompanied by appropriate legal and policy conservation measures.

#### Peru.

***Horizon 1 Findings:*** Horizon 1 describes the current situation in Peruvian peatlands. Distinctions were drawn between peatlands of the Amazon and the Andean regions. Peatlands of the Amazon were concentrated in large areas whereas in the Andes, the peatlands were small, fragmented and dispersed. In addition, there are distinctions between peatlands, wetlands and “black earth” or “terra preta” areas created with the charcoal from historic slash and burn practices, with limited data available to distinguish between them. There were differences too in terms of land tenure and property rights, with large areas of Amazonian peatlands owned by the state and to a lesser extent by local communities, while the Andean peatlands mainly owned by local communities.

Lack of effective governance was identified as a priority issue affecting both Andean and Amazonian peatlands. In particular, land tenure was identified as a key issue impacting the ability of local communities to govern peatlands. However, it was noted governance took place at local, regional, and international levels, and that “an ecosystem consciousness” was lacking across these different levels. In the Andes, for example, it was suggested that at the local level, ecosystem conservation was not typically a priority. In the Amazon, there is a lack of integration between national and regional governments to comply with effective peatland governance and policy as partly a consequence of new infrastructure projects.

Unsustainable use of resources was also identified as an issue, particularly in the Amazon where this is related to the unsustainable use of the *Mauritia flexuosa* palm swamps, locally known as *Aguajales*, where *M. flexuosa* fruits are used for subsistence and commercial purposes. Specifically, cutting of female *M. flexuosa* palms had left stands dominated by mainly male palms, leading to limited fruit production and species regeneration. Peat extraction in the Andes for compost production to sell in coastal regions was also mentioned as an example of unsustainable resource use in peatlands which is now banned.

Commercial agriculture was also identified as an issue, with Andean peatlands converted from extensive pasture to potato and maca production, or from semi-natural to improved grassland for livestock. In the Amazon, fires and coca production were not considered a major concern for the sustainability and resilience of peatlands, however oil palm and rice cultivation have targeted some peatlands. Mineral and oil extraction were also considered as threats to Peruvian peatlands (including illegal gold mining contaminating peatlands and an oil pipeline crossing peatlands).

***Horizon 3 Findings:*** Horizon 3 represents the vision for the future for Peruvian peatlands, as articulated by workshop participants. In this envisioned future:

- Peatlands, in all their different forms, are embedded in global consciousness and valued locally, regionally, nationally and globally for the role they have in sustaining the health of the planet.
- In Peru, an emphasis on good governance includes providing the means to protect peatland resources in the Amazon and the Andes. On the local level, governance is more explicit in terms of security of land tenure.
- Politicians are motivated to recognise the social, ecological, and cultural value of peatland ecosystems and provide governance for the overall system by employing a multisectoral approach.
- There is a shared understanding of peatlands’ social and ecological value and a commitment to participatory working practices, and “this social valuation is also necessary for enhanced ecological valuation”. Peatlands are understood and valued in the context of climate change.
- Enhanced knowledge exchange leads to better governance and policies that recognise the protection of peatlands as a priority. As knowledge is generated and shared, politicians are more motivated to recognise the value of peatland ecosystems.
- Local communities are financially supported to engage in conservation and best management practices. Well-developed systems of data collection and knowledge exchange inform decision making and feedback loops allow impacts to be monitored.

## Horizon 2 Findings:

Horizon 2 describes the transformational pathway through which Peruvian peatlands move from their current status (Horizon 1) to their envisioned future (Horizon 3). To facilitate change:

- In moving towards the third horizon, good governance is developed through the Peruvian government taking responsibility and assuming its role in planning and enforcing the new legislation on wetlands and peatlands. Participants felt that the Peruvian government had a catalysing role to play and that international support could help.
- Participants recognised the need to create enabling conditions that they described as “shifting the prevailing institutional logics or accepted norms, by changing established mindsets” through education and culture change. This includes being open to “more participatory working approaches that involve and include local populations and indigenous communities”. That said, there was recognition of the associated complexities and an acceptance that there may be multiple different perspectives that, in turn, bring questions of power and trust into consideration.
- They also argued that it is important to generate new scientific knowledge and value different knowledges, practices and traditions that communities have about peatland ecosystems and recognise that there are multiple different perspectives: “intergenerational transmission of knowledge is crucial”.
- Creating new finance opportunities that allow peatland conservation and management. For example, peatlands in Andean areas are integrated into carbon markets so that payment for their conservation is made to local communities, and in the Amazon, industrialisation of *M. flexuosa* fruit resource helps achieve sustainable harvest practices.

### Indonesia.

Indonesia’s peatlands are a vital ecosystem, estimated to hold between 55–57 billion metric tonnes of carbon [36]. Key findings from the business-to-business engagement process in Indonesia are outlined below:

- Although invites to interview were sent to companies across different industries operating in Indonesia, only one company outside of the palm sector responded. During discussions with palm oil companies, it also became apparent that despite multiple industries operating within and being reliant upon the same landscape, there were limited cross-sector initiatives working together to address peatland degradation and action is largely driven by the palm sector. Despite this, there were examples of effective collaborations within supply chains at peat dome level on integrated fire management. For example, the Siak Pelalawan Landscape Programme in Riau is driven by downstream private companies such as global food manufacturing companies like Unilever and PepsiCo, as well as upstream actors such as commodity traders, Cargill and Musim Mas. It was felt that this collaboration was largely a product of NGO attention in recent years being focussed specifically toward palm oil production in Indonesia.
- The actors we spoke to within the palm oil industry were primarily focussed on achieving zero-deforestation targets on peatlands. The urgency of meeting these targets was becoming increasingly pressing as target dates approached, and Deforestation Due Diligence legislation was being introduced within the European Union. These policies and commitments mean that palm planted on peatlands converted after 2008 are less likely to enter the supply chains of major traders, and incentives to convert peatland for palm production are therefore diminishing – if not reducing entirely. For companies operating on peat (via plantations or smallholder farms in their value chains), peat degradation will make the largest contribution to their land-based carbon emissions. As such, the narrow focus on net zero deforestation may detract from wider efforts to reduce peat-derived emissions. Given that these emissions are driven by the same factors as subsidence (i.e., by peatland drainage), emissions mitigation measures will also achieve peat subsidence mitigation.
- Whilst companies are focusing on achieving zero-deforestation in their own supply chains, the individuals we spoke to showed limited interest in or awareness of the material risks faced by peatland degradation in the landscapes in which they operate, such as stranded assets or the sinking of coastal peat soils and saltwater intrusion. There is a need to raise awareness not only of the temporal risks associated with peatland degradation and climate change (e.g., until the middle or the end of this century), but also of the spatial risks which will vary across plantation landscapes according to differences in depth of peat, drainage intensity, rate of subsidence, position in the landscape (top of peat dome versus edge of peat dome) and hence proximity to the drainage limit, but also including the spatial reach of their value chains through procuring fresh fruits from smallholders. Spatial risks will also vary with time as degradation and land subsidence proceed alongside the increasing impacts of a changing climate.

These results suggest considerable scope for landscape-scale initiatives involving both companies and communities and other actors with a stake in the management of peatland landscapes both now and over the coming decades. Whilst the primary engagement was with companies, these landscapes are also occupied and used by local communities, many of whom farm oil palm. In contrast, large-scale plantation businesses usually hold concessions on the shallow peat, as deep peat has been closed for plantation development since 1991 when all peat over 3 m was protected [80]. Given that smallholder expansion is driven by distance to roads and mills, better location of mills, design of transportation networks and government policies governing the locations and boundaries of concessions and migration settlements could slow, and even avoid, smallholder oil palm expansion into peat swamp forests. Improved enforcement of existing forestry laws could further conserve the remaining peat swamp forests. These findings present an opportunity for a landscape initiative that brings together different industries with a shared interest in peatlands in the Riau landscape, or indeed in other peat landscapes in Southeast Asia, with a focus on identifying and addressing material supply chain risks linked to peatland degradation. There would, however, be challenges in informing companies about these risks and generating interest in such a programme.

## Discussion

This paper presents the first application of the Three Horizons method to peatlands, providing evidence that this approach can integrate evidence from social and natural science literature alongside pre-workshop survey findings to inform facilitated discussion of transformational future pathways in three countries. The advice and support of local collaborators was vital to successful application of the framework, and its flexibility allowed the method to be adapted to both virtual and in-person workshop formats in very different social and cultural settings.

### Cross-country comparison

Across all regions, the Three Horizons method identified a need for integrated, multi-level governance approaches that recognize the value(s) of peatlands and involve local communities and other relevant parties in decision-making processes. In the Republic of Congo, the lack of specific legal protections for peatlands beyond Ramsar designations highlighted a significant governance gap. The Horizon 3 vision for a more positive future, where peatlands are better understood and protected, helped identify a need for enhanced national and transboundary legislation, cross-border cooperation, and the integration of local and scientific knowledge in both policy and governance of forested peatlands. The pathway through Horizon 2 emphasised ongoing investments in data collection, sustainable management, and the importance of collaboration and coordination between the Republic of Congo and the Democratic Republic of Congo, as called for by others at the Congo Basin level, e.g., [81,82].

Similarly, in the Democratic Republic of Congo, the challenges of extractive industries and the tension between economic development and peatland protection helped identify a need for comprehensive land use management plans, c.f. [82]. This is consistent with findings from [83] who demonstrated that logging concessions with an approved forest management plan have lower levels of deforestation in the Congo Basin. The Horizon 3 vision called for closer collaboration between the government and local communities, with an emphasis on recognizing the value of peatlands through national strategic planning. The pathway identified in Horizon 2 suggested the importance of land reform, community empowerment, and leveraging carbon markets for sustainable management. Although some commentators have suggested that there may be a conflict between REDD+ markets in the Congo Basin and community empowerment [84,85], others suggest that, managed appropriately, these markets may strengthen Congolese state legitimacy if coupled with land reform, enhancing community governance and capacity [86,87].

Many of these themes were echoed in Peru, where the distinction between Amazonian and Andean peatlands pointed to the need for tailored governance approaches that consider ecological, social, and economic differences between these regions. The envisioned future in Horizon 3 highlighted the importance of security of land tenure in effective governance, and multisectoral approaches that value the social, ecological, and cultural significance of peatlands. The transformational pathway through Horizon 2 focussed on more participatory approaches to peatland governance including educational approaches that capture different perspectives from within communities and facilitate intergenerational transmission of knowledge. There is a growing recognition of the need to foster epistemic justice in environmental governance, validating diverse knowledge systems and gaining valuable insights into environmental stewardship that are often overlooked by dominant Western epistemologies. For example, [88] and [89] argued for the recognition and integration of indigenous knowledge and voices into environmental governance as a way of addressing and redressing the historical marginalisation of non-Western forms of knowing. [90] further critiqued the extraction of Indigenous knowledge, challenging existing hierarchies that perpetuate environmental injustices and marginalise the epistemic contributions of local communities. Building on [91] call for a new “knowledge governance” framework, [90] advocated instead for Indigenous leadership in research, respecting the inherent sovereignty and rights of Indigenous peoples. For environmental governance to be just, equitable, and effective, it needs to be restructured around Indigenous voices and wisdom as autonomous and leading rather than ancillary and subordinate [92]. This shift is not only about integrating Indigenous knowledge into environmental governance but redefining knowledge governance itself to respect and protect Indigenous epistemologies and ways of being [93].

In all three countries where the Three Horizons method was applied, community empowerment and payments for ecosystem services emerged as key themes. For example, in DRC there was a focus on community engagement to shape and implement peatland science and policy, in the Republic of Congo there was an interest in engaging local communities in transboundary policy development and enforcement. In Peru discussions focussed around the potential for payment for ecosystem services schemes to fund local communities to manage peatlands more sustainably. This is consistent with scepticism reported by [43] about previous failed development interventions among peatland communities in the Loreto Region of the Peruvian Amazon, who were focussed more on pathways to economic empowerment, for example by developing markets for sustainable peatland products such as the *M. flexuosa* palm fruit (see also [47]).

Payment for ecosystem services was also a key theme emerging from interviews in Indonesia, although due to the focus of this work on businesses, there was a focus on corporate engagement at landscape scales to overcome free-rider effects and stimulate investment, rather than community engagement. There has been growing interest in community engagement in peatland restoration in recent years, with Indonesia’s “4R approach” to rewetting drained peat, revegetation of fragmented peatland, revitalization of local livelihoods [94,95] and reducing fire [96] being adapted to embed community engagement in each “R” whilst providing accountability via a fifth “R”, reporting and monitoring [97]. Community engagement in Indonesia has been driven in part by the need to tackle peat fires leading to transboundary pollution, which has required engagement with communities to modify traditional farming practices driving wildfire [98].

### Lessons from the three horizons method

As a theory-based approach to evaluating transformational future policy and governance pathways (c.f. [99]), Three Horizons has the capacity to identify causal pathways between actions, recognising, valuing and integrating diverse knowledge systems. For example, the DRC workshop identified the need to empower and engage local communities to shape and implement peatland policies in Horizon 3, which led to the identification of a number of near-term policy actions in Horizon 2, including the development of a national strategic plan to give local communities a platform to define and protect their rights, with policy support for land tenure reform and subsidies for sustainable management funded in part via carbon markets, which communities could be supported to access collectively through the creation of community-run forest reserves (see section 4.2.2).

The specificity and credibility of these policy proposals were in large part due to the engagement of both policy actors and local community representatives in the workshop, but the Three Horizons method provided a mechanism for structuring discussion and integrating ideas in a causally coherent transformation pathway. The integration of evidence from literature alongside pre-workshop survey findings helped workshop participants build on existing evidence, for example social science evidence of the different types of carbon markets applicable to DRC peatlands (reviewed in section 4.1) and natural science evidence of the location of forested peatlands that could be protected by REDD+ schemes [37,46].

The application of the Three Horizons method to the governance of tropical peatlands has identified options for addressing not just the visible, but also the conceptual and existential risks posed to peatlands by climate change, like the loss of peatlands to wildfire and subsidence (e.g., [75]). This approach, as applied across diverse contexts in Peru, the Congo Basin, and Indonesia, underscores the necessity of transcending conventional risk management strategies to contemplate deeper, systemic transformations in peatland governance. The findings advocate for a shift in focus from merely reacting to immediate threats, to proactively shaping resilient, sustainable futures for both peatlands and the communities that depend on them. As such, the Three Horizons methods may be applied in a range of environmental policy planning and evaluation contexts, where those formulating policy wish to value and manage complexity, and integrate multiple forms of formal and informal knowledge. However, integrating Indigenous knowledge into peatland policy and management remains challenging, and there is a need to recognize epistemological diversity and redistribute power to non-Eurocentric forms of wisdom [100,101].

There were also a number of challenges of implementing the Three Horizons method simultaneously in multiple countries and languages, in some cases involving online and/or remote engagement. The extent to which the evidence from the literature that was discussed during workshops differed between countries, with some focussing more on new inputs from participants. For example, in Peru, workshops focussed more on insights from previous social science fieldwork by the research team. Workshops were also significantly delayed, first due to the COVID-19 pandemic which made it too risky for researchers to travel to the field and facilitate gatherings in the Congo Basin. Further delays were caused by challenges transferring research funding to RoC and DRC and the global energy crisis which caused regular power cuts, disrupting email communication. As a result, workshops were held in capital cities in RoC and DRC, which limited representation from outlying regions.

## Conclusions

This paper sought to apply and evaluate the Three Horizons method to integrate research evidence with local knowledge to inform the transformation of tropical peatland governance in Peru, the Congo Basin, and Indonesia in response to threats from climate change. By integrating findings from the literature with insights from in-country workshop participants, the research identified a range of governance challenges and opportunities that span local, national, and international spheres. The narrative review underscored the necessity of a multifaceted approach to peatland governance, suggesting that no single policy or governance mechanism can fully address the complex challenges presented by climate change. Instead, there is a need for a mix of regulatory, financial, market mechanisms, partnerships, and co-management approaches, reflecting the diverse contexts and needs across different peatland regions. This research has underscored the urgent need for integrated governance strategies that not only counteract visible environmental threats but also tackle the underlying conceptual and existential challenges inherent in the current management and preservation of tropical peatlands.

By fostering inclusive and interdisciplinary dialogues that bridge traditional and scientific knowledge systems, the research highlights the potential for innovative governance models that are both adaptive and anticipatory in nature. These models promise not only to safeguard the ecological integrity of peatlands but also to secure the livelihoods and cultural heritage of the communities that depend on them. The findings suggest that achieving sustainable peatland governance requires a radical rethinking of current practices, advocating for a holistic approach that values diversity, promotes community empowerment, and leverages financial mechanisms for ecosystem services and building on indigenous structures and governance [102]. Future peatland policy and governance needs to protect peatlands from conversion and degradation by phasing out harmful activities and expanding protected areas, clarifying legal definitions, implementing gender-responsive policies, and leveraging financial and market instruments to incentivize sustainable management and restoration, while ensuring local communities play a central role and benefit from these initiatives.

The Three Horizons framework made it possible to assess the relevance of these alternative approaches for each country, alongside options arising from pre-workshop surveys from in-country participants, to envision the transformation of peatland governance from the current state to a more sustainable and resilient future. Through its application, it provided a structured basis that allowed us to compare the challenges and envisioned futures across a range of key global peatland settings. The application of this method illustrates the importance of integrating diverse sources of knowledge, including evidence from multiple disciplines and the lived experiences of those reliant on peatlands, to manage the complexity of social-ecological systems effectively. The method can help structure dialogues that identify causal pathways between actions, enabling the formulation of specific, credible policy proposals that incorporate local knowledge.
